# Evaluation of second-line apatinib plus irinotecan as a treatment for advanced gastric adenocarcinoma or gastroesophageal conjunction adenocarcinoma: a prospective, multicenter phase II trial

**DOI:** 10.3389/fonc.2023.1072943

**Published:** 2023-04-24

**Authors:** Jinglei Qu, Xin He, Ying Luo, Ping Yu, Ying Chen, Jing Liu, Xin Wang, Chang Wang, Tingting Liang, Yuxian Bai, Yu Han, Li Man, Chuanchun Leng, Caiyun Zhou, Lijie He, Xin Wang, Yunpeng Liu, Xiujuan Qu

**Affiliations:** ^1^ Department of Medical Oncology, The First Hospital of China Medical University, China Medical University, Shenyang, China; ^2^ Key Laboratory of Anticancer Drugs and Biotherapy of Liaoning Province, The First Hospital of China Medical University, Shenyang, China; ^3^ Liaoning Province Clinical Research Center for Cancer, Shenyang, China; ^4^ Clinical Cancer Research Center of Shenyang, The First Hospital of China Medical University, Shenyang, China; ^5^ Department of Oncology, Shengjing Hospital of China Medical University, Shenyang, China; ^6^ Department of Surgical Oncology and General Surgery, The First Hospital of China Medical University, Shenyang, China; ^7^ Cancer Center, The First Hospital of Jilin University, Changchun, China; ^8^ Department of Gastrointestinal Oncology, Harbin Medical University Cancer Hospital, Harbin, China; ^9^ Department of Medical Oncology, Anshan Cancer Hospital, Anshan, China; ^10^ Department of Medical Oncology, The Central Hospital of Anshan, Anshan, China; ^11^ The Fourth Oncology Departments, Huludao Central Hospital, Huludao, China; ^12^ Department of Medical Oncology, People’s Hospital of Liaoning Province, Shenyang, China; ^13^ Department of Medical Oncology, General Hospital of Benxi Iron and Steel Industry Group of Liaoning Health Industry Group, Benxi, China

**Keywords:** second-line apatinib plus irinotecan, gastric adenocarcinoma, gastroesophageal junction adenocarcinoma, treatment efficacy, safety

## Abstract

**Objective:**

Apatinib and irinotecan are used as systematic therapies for advanced gastric adenocarcinoma (GAC) and gastroesophageal junction adenocarcinoma (GEJA), while the evidence for their combination as second-line therapy in these patients is limited. This study aimed to evaluate the efficacy and safety of second-line apatinib plus irinotecan for the treatment of GAC and GEJA.

**Methods:**

In this prospective, multicenter phase II clinical study, 28 patients with advanced GAC or GEJA who received second-line apatinib plus irinotecan were recruited.

**Results:**

In total, 1 (3.6%) patient achieved complete response, 7 (25.0%) patients achieved partial response, 13 (46.4%) patients had stable disease, and 4 (14.3%) patients showed progressive disease, while clinical response was not evaluable or not assessed in 3 (10.7%) patients. The objective response rate and disease control rate were 28.6% and 75.0%, respectively. Meanwhile, the median (95% confidence interval (CI)) progression-free survival (PFS) was 4.5 (3.9-5.1) months, and the median (95% CI) overall survival (OS) was 11.3 (7.4-15.1) months. By multivariate Cox regression analysis, male sex, liver metastasis, and peritoneal metastasis were independently associated with worse PFS or OS, while treatment duration ≥5 months was independently associated with better OS. In terms of the safety profile, 89.3% of patients experienced treatment-emergent adverse events of any grade, among which 82.1% of patients had grade 1-2 adverse events and 64.3% of patients had grade 3-4 adverse events.

**Conclusion:**

Apatinib plus irinotecan as second-line therapy achieves a good treatment response and satisfactory survival with tolerable safety in patients with advanced GAC or GEJA.

## 1 Introduction

Gastric adenocarcinoma (GAC) and gastroesophageal junction adenocarcinoma (GEJA) are prevalent and dangerous malignancies worldwide, with more than 1 million newly diagnosed cases and over 0.7 million deaths in 2020 (1–3). It has been reported that the incidence of GAC has declined, while that of GEJA has steadily increased in recent years (4–6). Moreover, there is a large proportion of GAC and GEJA patients with advanced disease at diagnosis who lose the opportunity for potentially curative surgical resection (7, 8). According to the guidelines recommended by the Chinese Society of Clinical Oncology, the first-line systematic therapy for advanced GAC and GEJA is fluorouracil-based chemotherapy plus immunotherapy (combined with trastuzumab if the tumor presents human epidermal growth factor receptor 2 (HER2)-positive), while the second-line therapy is limited and merely includes paclitaxel, docetaxel, and irinotecan monotherapy or their combination, as well as paclitaxel and ramucirumab combination (9, 10).

Angiogenesis mediated by vascular endothelial growth factor (VEGF) has been identified as a key factor that facilitates the progression and metastasis of GAC and GEJA (11). Therefore, the application of antiangiogenic agents for the treatment of GAC and GEJA has received close attention. For instance, ramucirumab, a monoclonal antibody targeting VEGF receptor-2, has been approved by the Food and Drug Administration (FDA) for the treatment of advanced GAC and GEJA (12, 13). Meanwhile, apatinib, an oral tyrosine kinase inhibitor that also targets VEGF receptor-2, has been recommended as the third-line treatment for advanced GAC and GEJA in China since it dramatically prolongs progression-free survival in patients with advanced GAC and GEJA (9, 14). In the last two years, some clinical studies have shown that advanced GAC or GEJA patients who receive second-line apatinib plus chemotherapy present satisfactory response and survival, suggesting that apatinib plus chemotherapy may serve as a potential second-line therapy for advanced GAC and GEJA (14–21). In addition, a recent phase II study investigating ramucirumab plus irinotecan as second-line therapy in patients with advanced GAC also reveals the efficacy and safety of VEGF receptor-2 inhibitor plus irinotecan in those patients (22). However, more evidence is needed to facilitate the application of second-line apatinib plus chemotherapy for the treatment of advanced GAC or GEJA.

The current prospective, multicenter, phase II clinical study aimed to evaluate the treatment response, survival benefit, and adverse events of second-line apatinib plus irinotecan in advanced GAC or GEJA patients.

## 2 Methods

### 2.1 Participants

Between July 2017 and January 2022, 28 patients with advanced GAC or GEJA who received apatinib plus irinotecan as second-line treatment were recruited in this prospective, multicenter, single-arm, phase II clinical study. The inclusion criteria were as follows (1): 18-70 years old; (2) diagnosed with locally advanced or metastatic GAC/GEJA; (3) more than one measurable objective tumor lesion by spiral CT examination under Response Evaluation Criteria In Solid Tumors (RECIST) v1.1; (4) first-line chemotherapy failure before recruitment; (5) Eastern Cooperative Oncology Group performance status (ECOG PS) of 0 or 1; (6) acceptable hepatic and renal function; and (7) expected survival of more than 3 months. In consideration of safety, each subject should undergo UGT1A1*28 and UGT1A1*6 testing before enrollment. Patients with UGT1A1*28 (6/6) and *6 (G/G), UGT1A1*28 (6/6) and *6 (G/A), or UGT1A1*28 (6/7) and *6 (G/G) were eligible for enrollment. The exclusion criteria were (1) hypersensitivity to apatinib or irinotecan; (2) prior exposure to irinotecan or vascular endothelial growth factor receptor (VEGFR) inhibitors (such as apatinib, sorafenib, sunitinib); (3) uncontrolled hypertension (> 140/90 mmHg); (4) bleeding tendency; (5) received thrombolytics or anticoagulants; and (6) pregnant or lactating women. The study was approved by the First Hospital of China Medical University Institutional Review Board (No. 2016-197-10) with registration number NCT03116555 (https://clinicaltrials.gov). Each subject signed informed consent.

### 2.2 Treatment

Patients received oral apatinib (Jiangsu Hengrui Medicine, Jiangsu, China) 250 mg once daily every 3 weeks (as a treatment cycle). Irinotecan (Jiangsu Hengrui Medicine, Jiangsu, China) was administered 180 mg/m^2^ intravenously on the first day every 3 weeks (once every three weeks as a treatment cycle). In each treatment cycle, apatinib intermittent dose discontinuous were allowed if severe adverse events occurred, and patients would continue to treatment after the symptoms of adverse events disappeared or were ameliorated. The treatment was continued until progressive disease, the occurrence of intolerable toxicities, or the patient refused treatment.

### 2.3 Efficacy and safety assessments

The patients received radiographic evaluations every 6 weeks until progressive disease or intolerant toxicities. The treatment response was calculated through RECIST (v1.1). Each subject was closely followed up until death or lost to follow-up. The last follow-up date was June 1, 2022. The primary endpoint was progression-free survival (PFS), referring to the interval from treatment beginning to progressive disease or death. The secondary endpoints included overall survival (OS), objective response rate (ORR), and disease control rate (DCR). OS was defined as the interval from treatment beginning to death. The National Cancer Institute Common Terminology Criteria for Adverse Events (v4.0) was applied for grading adverse event severity.

### 2.4 Statistical analysis

The sample size was calculated *via* a hypothesis based on a previous study that the proportion of ORR was 26.9% with a confidence interval width of 0.369 (23). With a significance (α) level of 0.05, the minimum sample size was 25, adjusted to 28 for drop-out possibility. The statistical analyses were conducted using SPSS v27.0 (IBM Corp., USA), and the figures were created using GraphPad Prism v8.01 (GraphPad Software Inc., USA). PFS and OS are shown *via* Kaplan−Meier curves and were analyzed using the log-rank test. The survival analyses were performed *via* univariable and multivariable Cox regression analyses (backward stepwise methods; all factors were included). Comparison analyses were completed using the chi-square test or Fisher’s exact test. A *P* value <0.05 indicated statistical significance.

## 3 Results

### 3.1 Baseline characteristics

The patients had a mean age of 58.3 ± 7.5 years, consisting of 6 (21.4%) females and 22 (78.6%) males. There were 22 (78.6%) patients with tumors in the gastric and 6 (21.4%) patients with tumors in the gastroesophageal junction. Meanwhile, 7 (25.0%), 5 (17.9%), 5 (17.9%), 8 (28.6%), and 7 (25.0%) patients had tumor metastases on the liver, lung, peritoneum, lymph node, and others. In addition, 16 (57.1%) patients had <2 metastatic sites, while 12 (42.9%) patients had ≥2 metastatic sites. Regarding treatment history, 18 (64.3%) patients had a history of surgery; 20 (71.4%) patients received treatment <5 months, and 8 (28.6%) patients received treatment ≥5 months. Other baseline characteristics are specifically shown in **Table 1**. The specific previous first-line treatment regimens are shown in **Supplementary Table 1**.

**Table 1:** Baseline characteristics of patients.

Items	Patients (N = 28)
Age (years), mean ± SD	58.3 ± 7.5
Gender, n (%)	
Female	6 (21.4)
Male	22 (78.6)
ECOG PS, n (%)	
0	1 (3.6)
1	27 (96.4)
Site of primary tumor, n (%)	
Gastric	22 (78.6)
Gastroesophageal junction	6 (21.4)
Metastasis sites, n (%)	
Liver	7 (25.0)
Lung	5 (17.9)
Peritoneal	5 (17.9)
Lymph node	8 (28.6)
Others	7 (25.0)
Number of metastatic sites, n (%)	
<2	16 (57.1)
≥2	12 (42.9)
TNM stage, n (%)	
III	6 (21.4)
IV	22 (78.6)
History of surgery, n (%)	
No	10 (35.7)
Yes	18 (64.3)
Treatment duration, n (%)	
<5 months	20 (71.4)
≥5 months	8 (28.6)
Apatinib dosing suspension, n (%)	
No	23 (82.1)
Yes	5 (17.9)
Irinotecan dose adjustment, n (%)	
No	19 (67.9)
Yes	9 (32.1)

SD, standard deviation; ECOG PS, Eastern Cooperative Oncology Group performance status.

### 3.2 Treatment response

The median treatment duration of apatinib plus irinotecan was 3.8 months, with a range of 0.6-17.2 months. After treatment, clinical response was not evaluable or not assessed in 3 (10.7%) patients. There was 1 (3.6%) patient who achieved complete response (CR) and 7 (25.0%) patients who achieved partial response (PR). Meanwhile, 13 (46.4%) patients had stable disease (SD), and 4 (14.3%) patients showed progressive disease (PD). Therefore, the ORR was 28.6%, and the DCR was 75.0% (**Table 2**).

**Table 2:** Treatment response.

Items	Patients (N = 28)
Clinical response, n (%)	
CR	1 (3.6)
PR	7 (25.0)
SD	13 (46.4)
PD	4 (14.3)
Not evaluable or not assessed	3 (10.7)
ORR, n (%)	8 (28.6)
DCR, n (%)	21 (75.0)

CR, complete response; PR, partial response; SD, stable disease; PD, progressive disease; ORR, objective response rate; DCR, disease control rate.

### 3.3 PFS and OS

**Figure 1:**
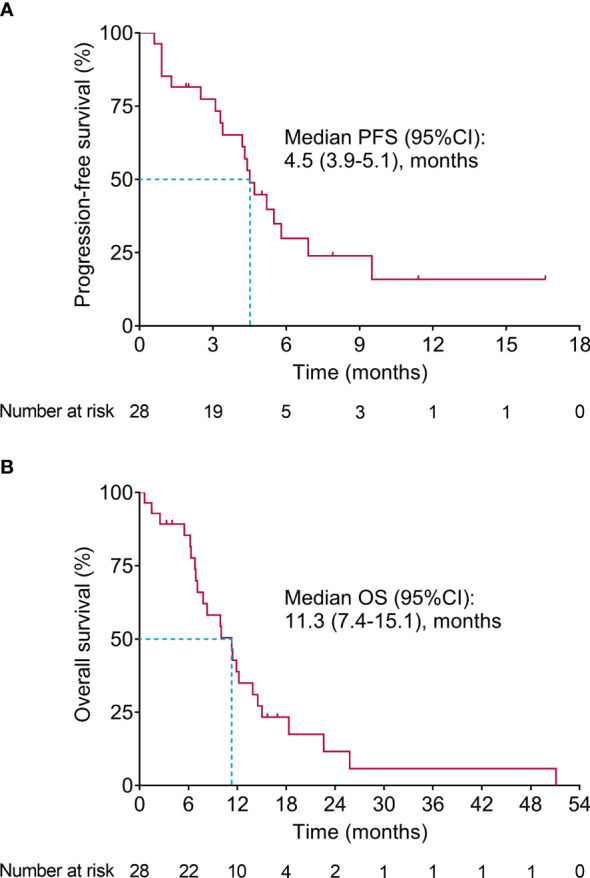
Survival. PFS **(A)** and OS **(B)** in advanced GAC or GEJA patients receiving second-line apatinib plus irinotecan.

Survival-related information was recorded, and PFS and OS were calculated to evaluate the long-term efficacy of second-line apatinib plus irinotecan. The data showed that the median (95% confidence interval (CI)) PFS was 4.5 (3.9-5.1) months (**Figure 1A**). Meanwhile, the median (95% CI) OS was 11.3 (7.4-15.1) months (**Figure 1B**). Moreover, apatinib intermittent dose suspension did not affect ORR, DCR, PFS, or OS (all *P*>0.05) (**Supplementary Table 2**).

### 3.4 Factors affecting PFS and OS

Univariate Cox regression analysis showed that gender (male *vs*. female) (*P*=0.020, hazard ratio (HR)=11.306) and liver metastasis (yes *vs*. no) (*P*=0.001, HR=7.375) were associated with worse PFS, while current treatment duration (≥5 months *vs*. <5 months) (*P*=0.007, HR=0.185) was associated with better PFS. Multivariate Cox regression analysis showed that gender (male *vs*. female) (*P*=0.004, HR=43.299), liver metastasis (yes *vs*. no) (*P*=0.005, HR=14.965), and peritoneal metastasis (yes *vs*. no) (*P*=0.002, HR=23.177) were independently associated with worse PFS, whereas current treatment duration (≥5 months *vs*. <5 months) (*P*=0.029, HR=0.043) was independently associated with better PFS (**Table 3**).

**Table 3:** Factors related to PFS by Cox’s proportional hazards regression analysis.

Items	*P* value	HR	95%CI	
			Lower	Upper
Univariate Cox’s regression analysis				
Age (≥60 years *vs*. <60 years)	0.418	0.688	0.278	1.700
Gender (Male *vs*. Female)	0.020	11.306	1.476	86.620
ECOG PS (1 *vs*. 0)	0.069	0.123	0.013	1.180
Site of primary tumor (Gastroesophageal junction *vs*. Gastric)	0.604	1.344	0.439	4.111
Liver metastasis (Yes *vs*. No)	0.001	7.375	2.211	24.600
Lung metastasis (Yes *vs*. No)	0.762	0.843	0.278	2.552
Peritoneal metastasis (Yes *vs*. No)	0.081	2.869	0.878	9.376
Lymph node metastasis (Yes *vs*. No)	0.821	0.886	0.311	2.526
Others metastasis (Yes *vs*. No)	0.514	0.711	0.255	1.983
Number of metastatic sites (≥2 *vs*. <2)	0.920	1.048	0.416	2.645
TNM stage (IV *vs*. III)	0.952	1.035	0.335	3.198
History of surgery (Yes *vs*. No)	0.674	0.816	0.316	2.107
Current treatment duration (≥5 months *vs*. <5 months)	0.007	0.185	0.055	0.626
Multivariate Cox’s regression analysis				
Gender (Male *vs*. Female)	0.004	43.299	3.281	571.374
Liver metastasis (Yes *vs*. No)	0.005	14.965	2.281	98.162
Peritoneal metastasis (Yes *vs*. No)	0.002	23.177	3.042	176.566
TNM stage (IV *vs*. III)	0.095	0.274	0.060	1.255
Current treatment duration (≥5 months *vs*. <5 months)	0.029	0.043	0.003	0.726

PFS, progression-free survival; HR, hazard ratio; CI, confidence interval; ECOG PS, Eastern Cooperative Oncology Group performance status.

In terms of OS, liver metastasis (yes *vs*. no) (*P*=0.001, HR=8.377) and peritoneal metastasis (yes *vs*. no) (*P*=0.021, HR=3.948) were associated with shorter OS. Further multivariate Cox regression analysis confirmed that liver metastasis (yes *vs*. no) (*P*<0.001, HR=30.140) and peritoneal metastasis (yes *vs*. no) (*P*=0.002, HR=14.600) were independently associated with unfavorable OS (**Table 4**).

**Table 4:** Factors related to OS by Cox’s proportional hazards regression analysis.

Items	*P* value	HR	95%CI	
			Lower	Upper
Univariate Cox’s regression analysis				
Age (≥60 years *vs*. <60 years)	0.484	1.351	0.581	3.143
Gender (Male *vs*. Female)	0.330	1.644	0.605	4.467
ECOG PS (1 *vs*. 0)	0.822	21.266	<0.001	7.9×10^12^
Site of primary tumor (Gastroesophageal junction *vs*. Gastric)	0.610	0.753	0.254	2.235
Liver metastasis (Yes *vs*. No)	0.001	8.377	2.262	31.024
Lung metastasis (Yes *vs*. No)	0.110	0.407	0.135	1.227
Peritoneal metastasis (Yes *vs*. No)	0.021	3.948	1.233	12.637
Lymph node metastasis (Yes *vs*. No)	0.732	0.845	0.322	2.218
Others metastasis (Yes *vs*. No)	0.367	0.631	0.232	1.715
Number of metastatic sites (≥2 *vs*. <2)	0.393	0.668	0.264	1.688
TNM stage (IV *vs*. III)	0.864	0.915	0.332	2.526
History of surgery (Yes *vs*. No)	0.537	0.764	0.325	1.796
Current treatment duration (≥5 months *vs*. <5 months)	0.495	0.733	0.299	1.792
Multivariate Cox’s regression analysis				
Liver metastasis (Yes *vs*. No)	<0.001	30.140	4.866	186.683
Peritoneal metastasis (Yes *vs*. No)	0.002	14.600	2.728	78.149

OS, overall survival; HR, hazard ratio; CI, confidence interval; ECOG PS, Eastern Cooperative Oncology Group performance status.

### 3.5 Adverse events

A total of 89.3% of patients experienced treatment-emergent adverse events of any grade, among which 82.1% of patients had grade 1-2 adverse events and 64.3% of patients had grade 3-4 adverse events.

Regarding hematological adverse events, the incidences of leukopenia, neutropenia, anemia, and thrombocytopenia of any grade were 60.7%, 60.7%, 50.0%, and 35.7%, respectively. Meanwhile, the incidences of grade 3-4 leukopenia, neutropenia, anemia, and thrombocytopenia were 28.6%, 46.4%, 14.3%, and 14.3%, respectively.

In terms of non-hematological adverse events, the most common ones of any grade were nausea (46.4%), elevated g-glutamyltransferase (GGT) (42.9%), diarrhea (42.9%), and fatigue (39.3%). In addition, grade 3-4 non-hematological adverse events were elevated GGT (14.3%), diarrhea (10.8%), elevated bilirubin (7.1%), elevated alanine aminotransferase (ALT) (3.5%), elevated aspartate aminotransferase (AST) (3.5%), hypertension (3.5%), and hand-foot syndrome (3.5%) (**Table 5**).

**Table 5:** Adverse events.

Items	Patients (N = 28), n (%)		
	All grade	Grade 1-2	Grade 3-4
Any patients with a treatment-emergent adverse event	25 (89.3)	23 (82.1)	18 (64.3)
Hematological adverse event			
Leukopenia	17 (60.7)	9 (32.1)	8 (28.6)
Neutropenia	17 (60.7)	4 (14.3)	13 (46.4)
Anemia	14 (50.0)	10 (35.7)	4 (14.3)
Thrombocytopenia	10 (35.7)	6 (21.4)	4 (14.3)
Non-hematological adverse event			
Nausea	13 (46.4)	13 (46.4)	0 (0.0)
Elevated GGT	12 (42.9)	8 (28.6)	4 (14.3)
Diarrhea	12 (42.9)	9 (32.1)	3 (10.8)
Fatigue	11 (39.3)	11 (39.3)	0 (0.0)
Elevated ALT	9 (32.1)	8 (28.6)	1 (3.5)
Elevated AST	9 (32.1)	8 (28.6)	1 (3.5)
Vomiting	9 (32.1)	9 (32.1)	0 (0.0)
Elevated bilirubin	6 (21.4)	4 (14.3)	2 (7.1)
Hypertension	6 (21.4)	5 (17.9)	1 (3.5)
Hand-foot syndrome	6 (21.4)	5 (17.9)	1 (3.5)
Proteinuria	6 (21.4)	6 (21.4)	0 (0.0)
Alopecia	6 (21.4)	6 (21.4)	0 (0.0)
Pyrexia	3 (10.7)	3 (10.7)	0 (0.0)
Elevated creatinine	2 (7.1)	2 (7.1)	0 (0.0)
Stomatitis	2 (7.1)	2 (7.1)	0 (0.0)
Anorexia	2 (7.1)	2 (7.1)	0 (0.0)
Neuropathy	2 (7.1)	2 (7.1)	0 (0.0)

GGT, g-glutamyltransferase; ALT, alanine aminotransferase; AST, aspartate aminotransferase.

## 4 Discussion

Irinotecan is one of the standard second-line chemotherapies for advanced GAC or GEJA, which could be metabolized into SN38, a DNA topoisomerase I inhibitor, thus suppressing the duplication of DNA and synthesis of RNA and exerting antitumor efficacy (24). Because of its spectral antitumor efficacy and wide application in digestive cancers, the current study chose irinotecan in combination with apatinib, a VEGFR-2 inhibitor that specifically represses angiogenesis to hinder tumor progression (25). In addition, a previous study suggested that irinotecan-based chemotherapy plus ramucirumab (another VEGFR-2 inhibitor) achieves a better treatment response than paclitaxel plus ramucirumab (26), which implied the potential superiority of irinotecan plus a VEGFR-2 inhibitor versus other chemotherapies plus a VEGFR-2 inhibitor. According to previous studies, the common dose of apatinib is 500 mg daily (15, 18, 19). Meanwhile, considering that advanced GAC or GEJA patients might be impossible to tolerate 150-180 mg/m^2^ irinotecan on the first day every 2 weeks, the dosage in our phase I clinical trial (data not published) was set as 500 mg apatinib daily and 180 mg/m^2^ irinotecan on the first day every 3 weeks (as a treatment cycle) to promote tolerability. However, all 3 patients experienced grade 3 adverse events, which mainly included diarrhea, hypertension, and granulocytopenia. The adverse events resulted in drug cessation in 2 of the 3 patients, and the dose of apatinib was reduced to 250 mg daily in the other patient. Therefore, in the current phase II study, the dose of apatinib was set as 250 mg daily and that of irinotecan was set as 180 mg/m^2^ on the first day every 3 weeks to increase the tolerability.

The application of second-line apatinib plus chemotherapy for the treatment of GAC and GEJA has become a hotspot in recent years. For instance, it has been reported that in patients with advanced GAC or GEJA refractory to first-line chemotherapy, the ORR is 21.6%, and the DCR is 83.8% (18). Meanwhile, a randomized, controlled trial revealed that second-line apatinib plus docetaxel achieved an ORR of 18.4% and DCR of 60.5% in advanced GCA or GEJA patients who failed first-line chemotherapy (17). Moreover, in advanced GAC patients who receive second-line apatinib plus chemotherapy (including docetaxel, paclitaxel, oxaliplatin, capecitabine, and tegafur), a total of 18.5% of patients achieve an objective response, and 92.6% of patients achieve disease control (15). In addition, a recent retrospective, real-world study investigated second-line or above apatinib plus irinotecan in patients with advanced gastric cancer (27). The current study was a prospective, multicenter, phase II clinical study focusing on second-line apatinib plus irinotecan in patients with advanced GAC or GEJA, which could further verify the therapeutic potential of second-line apatinib plus irinotecan for the treatment of these patients. The data revealed that the ORR and DCR were 28.6% and 75.0%, respectively, in advanced GAC or GEJA patients who received second-line apatinib plus irinotecan, which was within the range of previous study-reported ORR and DCR (15–21). In addition, the ORR and DCR were numerically higher than those in advanced GAC patients receiving second-line chemotherapy (19). A possible explanation could be that apatinib inhibited the progression of GAC and GEJA by suppressing angiogenesis, while chemotherapy directly killed tumor cells (24, 28). Therefore, the combination of these two antitumor agents with different mechanisms of performance might exert good effects on treating GAC and GEJA. Thus, the treatment response was acceptable in patients receiving second-line apatinib plus irinotecan. In the current study, 3 patients were excluded from treatment response evaluation. The reasons were as follows (1): The first patient was nonlocal. He/she failed to be followed up due to COVID-19, and the treatment response was not evaluated in this patient. (2) The second patient had poor drug consistency after 2 cycles of treatment. In addition, treatment response was not evaluated in this patient. (3) The third patient died after 1 cycle of treatment, which was considered to be associated with disease progression but not the drugs. Treatment response was not evaluated in this patient.

The survival of patients with advanced GAC or GEJA is quite unfavorable; it has been reported that the 5-year survival rate in these patients ranges from 5% to 30% (6, 29, 30), which is partly caused by the limited treatment choice after failure of first-line therapy. Previous studies have shown that second-line apatinib plus chemotherapy achieves certain survival in GAC or GEJA patients. For instance, in advanced GAC or GEJA patients receiving second-line apatinib plus chemotherapy (including docetaxel, paclitaxel, oxaliplatin, capecitabine, and tegafur), the median PFS and OS are 3.06 and 6.51 months, respectively (15). Meanwhile, in advanced GAC patients who receive second-line apatinib plus S-1, the median PFS and OS are 143.1 days (approximately 4.77 months) and 211.6 days (approximately 7.05 months), respectively (20). In the current phase II clinical study, it was observed that the median PFS and OS were 4.5 and 11.7 months, respectively, in advanced GAC or GEJA patients receiving second-line apatinib plus irinotecan. The PFS in the current study was within the range in previously reported studies, while the OS was numerically longer than that previously reported (15–21), which could be explained by the fact that some of the patients were locally advanced in our study and might have longer survival. Another possible explanation might be that compared with other studies, patients in the current study tended to have milder disease conditions and better performance status, such as younger age, lower ECOG PS score, and fewer metastatic sites, which contributed to a longer OS. Additionally, data from multivariate Cox regression analysis revealed that male sex, liver metastasis, and peritoneal metastasis were independent risk factors for worse PFS or OS, which were all well-recognized risk factors for mortality in advanced GAC or GEAJ patients in previous studies (31, 32). However, patients with these factors should not be excluded from the upcoming trial since these factors were prevalent in patients with advanced GAC or GEJA. Conversely, clinicians should pay close attention to patients with these factors to achieve better management in those patients.

According to previous reports, the hematological adverse events of apatinib plus chemotherapy mainly refer to leukopenia, neutropenia, anemia, and thrombocytopenia, whose incidences range from approximately 15% to 60% (14–21, 33). Regarding the non-hematological adverse events of apatinib plus chemotherapy, the common ones are fatigue, nausea and vomiting, elevated transaminase, diarrhea, etc., whose incidence ranges from approximately 30% to 75% (14–21, 33). The current phase II clinical study showed that the category of hematological and non-hematological adverse events was in line with previous studies; meanwhile, the incidences of these adverse events were also within the range of previous reports (14–21, 33). Meanwhile, the incidence of febrile neutropenia was 3 (10.7%). Growth factors were allowed for the treatment, but not for the prevention, of neutropenia. In addition, there were 12 patients who continued the original treatment regimen until the end of the study, 5 patients who stopped treatment due to treatment-emergent adverse events, and 11 patients with dose reduction due to treatment-emergent adverse events. These findings suggested that second-line apatinib plus irinotecan was tolerated in advanced GAC or GEJA patients.

This study might serve as vital evidence for the use of second-line apatinib plus irinotecan in patients with advanced GAC or GEJA, while some limitations of this study should be clarified. First, the sample size of this study was not large enough; further studies should verify our findings in a larger cohort. The possible reasons for slow accrual for the trial are listed as follows: a. most patients with GAC or GEJA were resectable, and the number of patients with advanced GAC or GEJA was small; b. with the continuously widening application of PD-1 inhibitors in China, an increasing number of patients were willing to receive PD-1 inhibitors. Therefore, the current study only enrolled 28 patients. Second, this study was a prospective, single-arm study; thus, further randomized controlled trials or meta-analyses might be needed to validate the efficacy and safety of second-line apatinib plus irinotecan. Third, the patients in this study received different first-line treatments, which could be a potential confounding factor. Fourth, the underlying mechanisms of apatinib plus irinotecan in inhibiting GAC or GEJA should be further explored by *in vitro* and *in vivo* experiments.

Collectively, apatinib plus irinotecan as second-line therapy achieves a good treatment response and satisfactory survival with tolerable safety in patients with advanced GAC or GEJA, which could serve as a potential treatment option for these patients. However, these findings should be further verified in studies with larger sample sizes to provide more reliable evidence to support second-line apatinib plus irinotecan in patients with advanced GAC or GEJA.

## Data availability statement

The original contributions presented in the study are included in the article/**Supplementary Material**. Further inquiries can be directed to the corresponding authors.

## Ethics statement

The studies involving human participants were reviewed and approved by the First Hospital of China Medical University Institutional Review Board (No. 2016-197-10) with the registration number NCT03116555 (https://clinicaltrials.gov). The patients/participants provided their written informed consent to participate in this study.

## Author contributions

XQ and YLi contributed to the study conception and design. Material preparation was performed by JQ, XH and YLu. Data collection was performed by PY, YC and JL. Data analysis was performed by XW (7th author), CW and TL. YB, YH and LM were responsible for the interpretation of the data. The first draft of the manuscript was written by CL and CZ. LH and XW (16 author) contributed to manuscript revisions of the intellectual content. All authors contributed to the article and approved the submitted version.
