# Supplementary material for: Evaluation of second-line apatinib plus irinotecan as a treatment for advanced gastric adenocarcinoma or gastroesophageal conjunction adenocarcinoma: a prospective, multicenter phase II trial
Source: Front Oncol. 2023 Apr 24;13:1072943. doi: 10.3389/fonc.2023.1072943 (PMC10166633; doi:10.3389/fonc.2023.1072943)
Supplement: Supplementary file 1 [file DataSheet_1.docx]

***Supplementary Materials***

**Supplementary Table 1.** First-line treatment regimens.

| First-line treatment regimens | Patients (N = 28) |
| --- | --- |
| SOX | 11 (39.3) |
| XELOX | 7 (25.0) |
| TS | 2 (7.1) |
| Others | 8 (28.6) |

SOX, S-1 plus oxaliplatin; XELOX, capecitabine plus oxaliplatin; TS, thymidylate synthase.

**Supplementary Table 2.** Correlation of apatinib dose suspension with outcomes.

| Items | Apatinib dose suspension | | *P* value |
| --- | --- | --- | --- |
|  | No | Yes |  |
| ORR, No. (%) |  |  | 0.606 |
| No | 17 (73.9) | 3 (60.0) |  |
| Yes | 6 (26.1) | 2 (40.0) |  |
| DCR, No. (%) |  |  | 1.000 |
| No | 6 (26.1) | 1 (20.0) |  |
| Yes | 17 (73.9) | 4 (80.0) |  |
| PFS (months), median (95%CI) | 4.5 (3.2-6.0) | 4.7 (3.9-5.5) | 0.614 |
| OS (months), median (95%CI) | 10.1 (5.3-14.9) | 12.3 (5.2-19.5) | 0.221 |

ORR, objective response rate; DCR, disease control rate; PFS, progression-free survival; CI, confidence interval; OS, overall survival.
